# The Landscape of Mobile Apps for Healthy Eating: Case Study for a Systematic Review and Quality Assessment

**DOI:** 10.2196/68737

**Published:** 2026-01-30

**Authors:** Garlene Zamora Zamorano, Alejandro Déniz-García, Alezandra Torres-Castaño, María Luisa Álvarez-Malé, Inger Torhild Gram, Guri Skeie, Ana M Wägner

**Affiliations:** 1 Instituto de Investigaciones Biomédicas y Sanitarias Universidad de Las Palmas de Gran Canaria Las Palmas de Gran Canaria Spain; 2 Department of Endocrinology and Nutrition Complejo Hospitalario Universitario Insular Materno-Infantil de Canarias Instituto de Investigaciones Sanitarias de Canarias Las Palmas de Gran Canaria Spain; 3 Evaluation Unit of the Canary Islands Health Service Instituto de Investigación Sanitaria de Canarias Tenerife Spain; 4 Norwegian Centre for eHealth Research University Hospital of North Norway Tromsø Norway; 5 Department of Community Medicine Faculty of Health Sciences UiT the Arctic University of Norway Tromsø Norway; 6 Nutrition and Metabolism Branch International Agency for Research on Cancer Lyon France

**Keywords:** apps, applications, mobile health, mHealth, nutrition, healthy eating, eHealth, digital health

## Abstract

**Background:**

Mobile apps are being increasingly used to foster healthy lifestyles. There is a growing need for clear, standardized guidelines to help users select safe and effective health apps.

**Objective:**

Our study aimed to highlight the importance of establishing a structured framework for quality evaluation in mobile health (mHealth) through a case study of mobile apps promoting healthy eating.

**Methods:**

We conducted a systematic review of apps promoting healthy eating that had already been evaluated by one or more of 28 recognized health app certification bodies. Three rounds of app evaluations were conducted by experts in nutrition and behavior change. The first two rounds focused on the quality of the content of the recommendations and were performed pairwise using the Quality Evaluation Scoring Tool (QUEST), which has not been previously used by the certification bodies. In addition, in the second and third rounds, each reviewer answered the question “How probable is it that you would recommend this app?” using a subjective scale score from 0 to 10. In the third round, this score was weighed by usability (30%), content quality (40%), and promotion of behavior change (30%). Discussions were held to resolve scoring discrepancies and to identify the top-quality apps. We also assessed correlations among QUEST, Google Play Store, and certification body scores.

**Results:**

Of the 41 apps identified by five certification bodies, 19 (46.3%) met the inclusion criteria and were examined. Only 16 (84.2%) of these remained accessible for the second round. Eight of these surpassed 20 points (out of a maximum of 28) on the QUEST scale and were evaluated by all six experts in the third round, and the top 5 (62.5%) apps were selected. No correlations were found among QUEST, Google Play Store, and certification body scores.

**Conclusions:**

Despite numerous evaluations by various certification bodies, only 5 (12.2%) of the 41 apps met the quality standards set by our experts. Our results mark the importance of rigorous, transparent, and standardized app evaluation processes to guide users toward making informed decisions about health apps. Guidelines for developers for the design of evidence-based, unbiased, high-quality apps, as well as technological solutions for real-time monitoring of the health apps, would address these challenges and improve reliability.

## Introduction

As our world becomes increasingly digital, the use of mobile apps for health-related purposes (health apps) is on the rise [1,2]. The World Health Organization’s (WHO) Global Observatory of eHealth describes health apps as key components of the broader domain of mobile health (mHealth). The use of mobile devices (eg, smartphones, patient-monitoring tools, personal digital assistants, and other wireless devices) is instrumental to support medical and public health practices. eHealth encompasses the cost-effective and secure use of information and communication technologies for health care and related fields [3]. Since WHO’s acknowledgment in 2016 [4], the market has expanded rapidly. There has been an exponential increase in health app availability from 325,000 in 2017 [5] to an estimated 255 billion app downloads in 2022 [1,2].

Despite this growth, health care professionals lack unified standards for evaluating health app quality, safety, and effectiveness [6]. Recent European Union regulations on medical devices, such as Regulation (EU) 2017/745 enacted in May 2021, represent significant development. These regulations classify certain health apps as medical devices, necessitating adherence to specific criteria for approval. Supplementary documents from the European Commission [7,8] complement these regulations. The regulations also establish a medical device database to enhance transparency for both patients and health care providers. However, despite mHealth apps being within the medical device framework and subject to all corresponding laws at the European level, there are pending challenges related to user data management. Commercial platforms typically do not provide reliable information regarding the efficacy or safety of the apps they offer. Health apps should be substantiated by scientific evidence, facilitating their endorsement by health care professionals and enabling end users to benefit from a validated certification system [9,10], which should include the assessment of not only the quality of the information provided but also other relevant factors associated with app quality, such as usability and behavior change promotion [11].

This case study proposed a robust process for the evaluation of health apps, using dietary intervention apps promoting healthy eating as an example, to illustrate both the evaluation method and the challenges faced by end users and health care prescribers. A particular focus was placed on the quality of the health information provided by the apps.

## Methods

### Study Design

Our approach was structured in two stages: first, the systematic identification of relevant apps and, second, their assessment based on predefined quality, usability, and evidence-based criteria. This sequence aims to be a realistic, reproducible way to support both health care professionals and end users in the navigation and evaluation of the growing mHealth landscape.

### App Identification

An initial exploratory analysis was conducted to identify projects, initiatives, and organizations involved in the evaluation of health apps. Most of these resources have been catalogued in two documents by the Spanish Ministry of Health [12] and the European project mHealth-Hub [13,14]. Both include governmental and nongovernmental efforts. Each initiative was then individually reviewed to examine its content and structure. Notable heterogeneity among the initiatives was observed, allowing them to be classified into two main groups: (1) platforms offering pre-evaluated apps, used as databases for the search, and (2) those focused on normative aspects, standardization, and quality assessment. Given the diversity in the approaches and structures of these platforms, the search began with general terms related to the study’s focus, such as healthy eating, staying healthy, diet, lifestyle, preventive medicine, weight or healthy habits, which were later adapted to the navigation logic, taxonomy, or filtering system of each initiative (for more details, see Multimedia Appendix 1).

From August to December 2021, we searched for health apps that met the following inclusion criteria: aimed at adults (over 18 years old), available in English or Spanish, and offering nutritional guidance (eg, recommendations for dietary changes or meal planning). Apps that solely functioned as food diaries, calorie counters, or barcode readers but did not include any specific recommendations were excluded. We examined app descriptions and features to confirm eligibility.

In the absence of established guidelines for health app evaluation, we followed the Preferred Reporting Items for Systematic Reviews and Meta-Analyses (PRISMA) checklist (Multimedia Appendix 2) [15,16] adapted to our research objectives. We reviewed 28 certification bodies (see Table S1 in Multimedia Appendix 1), of which only 5 (17.9%) contained apps meeting our inclusion criteria (see Table 1 for additional details):

**Table 1:** Summary of certification/assessment bodies that included apps promoting healthy eating.

Name	Year it started	Country	Reviewers	Evaluation criteria	Public or private
MyHealthApps^a^	2013	United Kingdom	End users, associations, or caretakers	Scoring system	Private
Healthy Living Apps Guide	2018	Australia	Expert reviewers (at least two on behavior change/public health)	MARS^b^ (functionality) and ABACUS^c^ (behavior change)	Public (Government of Victoria)
ORCHA^d^	2018	United Kingdom	Experts and reviewers (end users considered)	A 7-step system	Private (cooperation with the NHS^e^)
GGD^f^ AppStore	2016	Netherlands	Experts and end users	A 4-step assessment based on questionnaires, including a final evaluation of behavior change techniques used	Public (GGD GHOR^g^)
Health Navigator	2017	New Zealand	Experts, clinical reviewers, and end users	Internal revision by experts, clinical revision, and end-user revision.	Public Ministry of Health

^a^Closed on May 17, 2022.

^b^MARS: Mobile App Rating Scale.

^c^ABACUS: App Behavior Change Scale.

^d^ORCHA: Organization for the Review of Care and Health Applications.

^e^NHS: National Health Service.

^f^GGD: *Gemeentelijke Gezondheidsdienst* (Municipal Health Service).

^g^GHOR: Geneeskundige Hulpverleningsorganisatie in de Regio (Regional Medical Emergency Preparedness and Planning).

*Healthy Living AppsGuide* [17] assesses app functionality using the Mobile App Rating Scale (MARS) [18] and behavior change using the App Behavior Change Scale (ABACUS) [19]. Each criterion is rated on a scale of 0-5 stars, with additional considerations, such as app cost and data export capabilities.

*MyHealthApps* [20] focused on user preferences and developer-related data. This resource operated as a catalog, rather than assigning individual scores to apps. It assessed transparency regarding pricing, contact details, geographical location, and security measures. Unfortunately, it closed in May 2022.

*ORCHA* (Organization for the Review of Care and Health Applications) [21] uses a seven-step evaluation system comprising three main domains: data, professional guarantee, and usability and accessibility. App functionalities and features are also considered. A maximum score of 100 points is attainable for each domain, with ratings above 65 considered good quality. Scores between 45 and 65 suggest areas for further investigation, and scores below 45 deem the app (or domain) potentially unsafe or ineffective.

*Gemeentelijke Gezondheidsdienst* (*GGD*) *AppStore* [22] follows an evaluation method considering app availability, pricing, compatibility with different operating systems, provider contact details, promotion of healthy behavior, data management, and goal setting. To enter evaluation, apps must focus on self-care and include at least two methods for behavior change. The apps are also assessed for usability, reliability, privacy, safety, and relevance, with each characteristic rated as good, sufficient, or inadequate. Apps are scored up to a maximum of 5 stars.

*Health Navigator* [23] is supported by the New Zealand Ministry of Health and provides a library of reliable apps. The evaluation process encompasses app features, functionalities, quality of information, and relevance to users. Apps must fulfil specific criteria, including evidence-based content and an evaluation of effectiveness, acceptability, and usability, and include a privacy statement. Clinicians with diverse expertise rate the apps from 1 (very poor, not recommended) to a maximum of 5 stars (excellent). End users also provide feedback. Finally, apps deemed clinically unsafe or potentially harmful are excluded.

**Figure 1:**
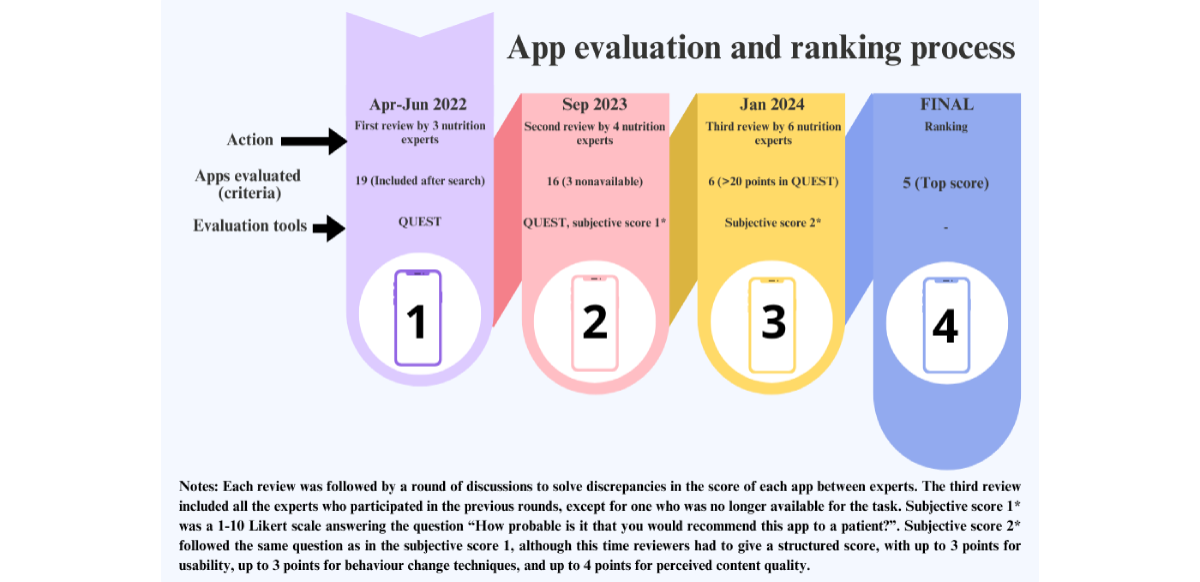
Diagram depicting the evaluation process of the apps.

As health apps are frequently updated, a follow-up search was performed in May 2022. By that time, *MyHealthApps* was no longer active. From this point on, three rounds of app evaluations followed (see Figure 1).

### App Assessment

The apps underwent three rounds of evaluation involving a total of seven reviewers who downloaded and assessed them on the devices described later. An endocrinologist (specialized in endocrinology and nutrition), one dietician, two psychologists, and three epidemiology and public health professionals participated. A second endocrinologist led the evaluation process and coordinated and summarized meetings.

#### Evaluation Round 1 (April-July 2022)

The content of each of the selected apps was evaluated by pairs from a panel of three nutrition experts (author AD-G, specialized in endocrinology and nutrition; author GZZ, a registered dietician; and Cristina Ruano Rodríguez, a lecturer in nutrition and public health). To facilitate comparison across the apps, we used the Quality Evaluation Scoring Tool (QUEST) [24], not used by any of the evaluating bodies that included the selected apps. QUEST was originally designed to assess the quality of health information in digital media and has been tested for reliability and validity. It offers a total score ranging from 0 to 28 points based on the following criteria: Authorship (0, 1, or 2 points) evaluates the ease of identifying the authors of the content; attribution (0, 3, 6, or 9 points) assesses whether health information is backed by scientific studies; type of study (0, 1, or 2 points, if at least 6 points have been given to *attribution*) evaluates the studies’ quality; conflict of interest (0, 3, or 6 points) evaluates whether the information promotes purchase of products or services; complementarity (0 or 1 point) evaluates whether the information supports the health provider-patient relationship; and tone (0, 3, or 6 points) assesses language used as biased, neutral, or acknowledging the limits of knowledge.

The apps were downloaded by the reviewers to Android devices (two Galaxy Tab A7 Lite SM-T220 running on One UI Core 3.1 and Android 11 and a Redmi 9A M2006C3LG MIUI Global 12.0.20 mobile phone running on MIUI Global 12.0.20 Estable [QCDEUXM] and Android 10QP1A.190711.020). Whenever a premium version was available, it also was tested. A data extraction template was developed, including reviewer ID, date of the evaluation, name of the app, developer, version, device where it was downloaded, score for each item in QUEST, and comments. The mean total score was calculated to rank the apps, and inter-reviewer agreement for each QUEST item was measured using weighted κ coefficients. Concordance for the total score was evaluated using the intraclass correlation coefficient. We also explored potential correlations between the QUEST scores, Google Play Store ratings, and the scores from each assessment body (all three, nonnormally distributed quantitative continuous variables) using Spearman’s coefficients. For these procedures, we used the *vcd* library in R software (RStudio version 1.3.1056, R Foundation for Statistical Computing). Following the analysis, discrepancies in the evaluation of QUEST items were discussed to identify their underlying causes.

#### Evaluation Round 2 (September 2023)

An expanded panel of four additional reviewers (authors AT-C and MLA-M, psychologists, experts in behavior change; author ITG, professor in epidemiology, expert in health promotion; and author GS, public health nutritionist, expert in prevention through diet) re-evaluated the apps (4-5 each) using the same methodology but limiting the time spent in the evaluation to a maximum of 45 minutes per app. Apps with a score average over 20 points (out of a maximum of 28) either in the initial evaluation or in the update were selected for further analysis. Although no given cutoff is recommended for the tool, this threshold was chosen to ensure the inclusion of apps that meet a fair-to-high standard. All evaluators were asked to subjectively score (0-10 points) their assigned apps by answering the question “How probable is it that you would recommend this app?” One of the reviewers (AD-G) scored all of them. Apps with a difference of 3 or more points in the initial and updated QUEST scores were discussed within the same reviewer pair (one of the reviewers involved in the first round and the new expert involved in the second round) to solve or explain this discrepancy. This process was documented, summarized, and then discussed during a meeting involving all the authors, including author AMW (specialist in endocrinology, nutrition, and diabetes). The meeting was recorded, transcribed, and summarized in a document, which was shared with the participants for feedback.

#### Evaluation Round 3 (January 2024)

The last version of the selected apps was downloaded and assessed by six experts (GZZ, AD-G, AT-C, MLA-M, ITG, and GS), who had participated in the previous rounds. A subjective score was given again, this time assigning up to 4 points for content quality (this aspect being the most relevant for our research), 3 for usability, and 3 for promoting behavior change. The specific question to answer in this round was “How probable is it that you would recommend this app for someone to improve their healthy diet, based on the quality of the content (40%), usability (30%), and promotion of behavior change (30%)?”

Further discussion among all the authors led to the final ranking and the selection of the top 5 apps.

### Ethical Considerations

Given the nature of this study, no ethics committee approval or informed consent to participate was needed. All authors declared their consent for publication.

## Results

### App Details

Of the 28 certification bodies identified (see Table S1 in Multimedia Appendix 1), 14 (50%) catalogued a total of 557 apps across various categories, encompassing terms such as “healthy eating,” “nutrition,” “diet”, “healthy practices,” and “lifestyle”. Only 5 (17.9%) included apps that fulfilled our inclusion criteria (see Table 1). These certification bodies used a variety of criteria in their evaluation processes, in terms of both the elements assessed and the tools used. Nevertheless, there was a significant overlap in the factors considered key for determining app quality, including functionality, usability, engagement, aesthetics, privacy, data protection, and effectiveness in promoting behavior change.

**Figure 2:**
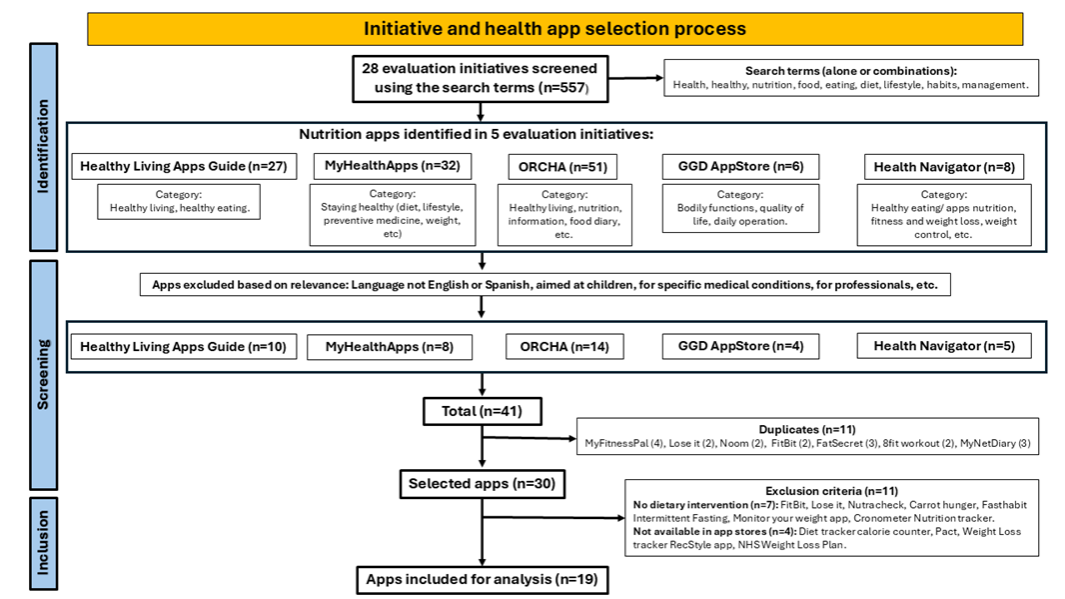
PRISMA 2020 flowchart describing app search and findings. GGD: *Gemeentelijke Gezondheidsdienst* (Municipal Health Service); ORCHA: Organization for the Review of Care and Health Applications; PRISMA: Preferred Reporting Items for Systematic Reviews and Meta-Analyses.

A total of 41 (7.4%) apps met the inclusion criteria. After eliminating duplicates, 30 (73.2%) apps remained, of which 11 (36.7%) met the exclusion criteria (see Figure 2).

The characteristics of the selected 19 (63.3%) apps are summarized in Table 2. Of these 19 apps, 5 (26.3%) were included in two or more of the certification bodies: *MyFitnessPal*, *FatSecret*, *MyNetDiary*, *Noom*, and *8fit Workout & Meal Planner*. Only the apps included in ORCHA had undergone formal evaluation of their content.

As per the definition, all apps included nutritional interventions, but most also combined different strategies or features, making their categorization challenging. Overall, 14 (73.7%) apps included weight control or tracking (numbered 1-6, 8, 11, 12, 14, 16-19 in Table 2), 12 (63.2%) allowed for calorie counting (numbered 1-4, 6-8, 11-13, 17, 19 in Table 2), and 13 (68.4%) facilitated the recording of physical activity (numbered 1-4, 6-9, 14-17, 19 in Table 2). Furthermore, 12 (63.2%) apps (numbered 1-8, 10, 11, 16, 18 in Table 2) incorporated behavior change techniques, such as motivational feedback, health education promotion, and goal setting.

**Table 2:** Main features of the 19 selected and evaluated health apps.

App number	App name; developer	Description
1	Noom; Noom Inc	It uses behavior change psychology to promote healthier habits, weight loss, and health goals. It can track meals and link to a pedometer.
2	YAZIO; YAZIO	It is a food diary and calorie counter that also offers diets and recipes, including vegan, vegetarian, and intermittent fasting options.
3	FatSecret; FatSecret	It is a food diary and calorie counter. It allows the user to scan products barcodes, tracking weight and keeping a record of meals and consumed food photos.
4	MyNetDiary; MyNetDiary Inc	It is a food log and calorie counter that also provides diets and recipes. It has a food database and a barcode scanner. It also provides motivational messages to achieve nutritional goals.
5	DietBet; WayBetter Inc	It aims to help the user achieve healthy weight loss and maintain motivation. It offers a support community, customer service, and expert-led games (coaches, nutritionists, etc).
6	MyFitnessPal; MyFitnessPal Inc	It offers recipes, a meal planner, a calorie counter, and workout plans. It also allows tracking progress and setting macronutrient goals.
7	MyPlate^a^; LIVESTRONG	It allows tracking calories and exercises. It also tracks the user’s progress based on calorie and nutrient goals and provides motivation through support groups.
8	LIFESUM; Lifesum	It functions as a food diary and offers various types of diets and recipes. It also allows tracking exercise and water intake and setting weight goals and health data.
9	8fit Workout & Meal Planner; Urbanite Inc	It offers workout routines in different categories (boxing, yoga, high-intensity interval training, etc), along with meal plans.
10	Freeletics Nutrition; Freeletics GmbH	It acts as a nutrition coach to help the user achieve dietary goals. It guides the user toward healthy eating, recipes, etc, and helps them adapt nutrition to personal goals.
11	GetFit-Daily Meal Planner^a^ App Prodakshn;OOO	It provides daily and weekly meal plans to lose, maintain, or gain weight based on the user’s goals. It also calculates nutrients, calories, water intake, BMI, etc, and includes reminders.
12	HealthifyMe; HealthifyMe	It can act as a calorie counter, provides weight loss and exercise plans, guides sleep hygiene, etc.
13	Eat This Much-Meal Planner; Eat This Much Inc	It is a meal planner based on dietary preferences, budget, etc, and can be configured for different types of diets.
14	LIFE Extend^a^; LifeOmic	It is marketed as a precision health mobile app to help improve users’ health using five pillars of health.
15	Fastic Fasting Ap; Fastic GmbH	It promotes intermittent fasting with different levels of intensity (gentle, moderate, intense).
16	Second Nature; Second Nature	It provides a lifestyle change program that helps lose weight and develop healthy habits. Nutritionists guide the user throughout the program. The app also includes a support group.
17	Uplyfe-Precision Nutrition^a^; Uplyfe AG	It is marketed as a customized nutrition and lifestyle change app-guided program based on scientific findings. It also provides personalized nutrition plans, activities, and symptom tracking.
18	Freshwell; Freshwell	Health care personnel promote a low-carb diet. The app provides informative weekly modules, a meal planner, etc.
19	Contador de calorías; Virtuagym	It provides a personalized nutrition plan according to the user’s lifestyle and goals.

^a^No longer available.

### App Evaluation

Table 3 presents the scores given to each app by the reviewers during the three rounds of assessment, by the certification bodies, and by a commercial app store.

**Table 3:** Scores by the different evaluation tools and rounds of revision for each app.

Evaluation rounds and app (name; first version evaluated; date of update of that version; year launched)			Scores																
			Round 1: QUEST^a^		Round 2: QUEST		Round 2: subjective		Round 3: subjective		Google Play Store (stars); August 19, 2022		ORCHA^b^		Health Navigator		GGD^c^ AppStore		Healthy Living Apps Guide
Maximum score			28		28		10		10		5		100		5		5		5
Number of evaluators (total, per app)			3, 2		4, 1		6, 6		6, 6		—^d^		—		—		—		—
**Apps that went through all evaluation rounds**																			
	Freshwell; 1.1.1; October 25, 2021; 2021	21.5		28		7.2		6.9		5		68		—		—		—	
	Yazio; 7.10.8; August 8, 2022; 2014	21.5		18		8.2		7.4		4.6		—		—		4.1		—	
	LIFESUM; 11.0.0; June 30, 2022; 2011	20.5		14		7.2		6.8		4.5		—		—		—		—	
	MyNetDiary; 8.1.1; August 12, 2022; 2010	18.5		22		6.2		6.8		4.6		79		—		—		2.5	
	Second Nature; 6.10.2; August 11, 2022; 2016	12		28		8		8.5		4.6		84		—		—		—	
	Freeletics Nutrition; 1.27.12; January 27, 2020; 2016	7.5		21		6.7		5.5		3.8		—		—		—		2.5	
**Apps included in the first two rounds only**																			
	HealthifyMe; 18.7.1; August 1, 2022; 2013	21.5		13		2.5		—		4.4		—		—		—		2	
	LIFE Extend^e^; 5.8.3; August 9, 2022; 2019	20.5		24		5.5		—		4.1		74		—		—		—	
	Noom; 10.28.0; August 17, 2022; 2011	17.5		12		—		—		4.2		80		5		—		—	
	Fastic Fasting App; 1.105.0; August 16, 2022; 2019	14		11		—		—		4.7		70		—		—		—	
	Fatsecret; 9.12.5.2; May 25, 2022; 2007	11		10		—		—		4.7		—		4		4		—	
	Eat This Much-Meal Planner; 2.0.12; August 12, 2022; 2016	11		13		—		—		4.4		—		—		—		2	
	8fit Workout & Meal Planner; 22.4.0; May 9, 2022; 2014	10		14		—		—		4.3		75		—		—		2.5	
	DietBet; 18.0.0; August 4, 2022; 2014	8.5		6		—		—		4.7		—		—		—		2.5	
	MyFitnessPal; 22.15.0; August 3, 2022; 2010	6.5		5		—		—		4.4		47		3		3		—	
	Contador de calorías; 3.7.3; April 28, 2022; 2014	6		6		—		—		4.5		—		—		4.5		—	
**Apps included in the first round only**																			
	GetFit-Daily Meal Planner ^e^; 1.3.4; April 23, 2023; 2020	16.5		—		—		—		1		—		—		—		2.5	
	MyPlate^e^; 3.5.3(63); September 30, 2021; 2015	14		—		—		—		4.6		—		—		—		—	
	Uplyfe-Precision Nutrition^e^; 1.8.1; September 21, 2021; 2020	13.5		—		—		—		4.2		70		—		—		—	

^a^QUEST: Quality Evaluation Scoring Tool.

^b^ORCHA: Organization for the Review of Care and Health Applications.

^c^GGD: *Gemeentelijke Gezondheidsdienst* (Municipal Health Service).

^d^Not applicable.

^e^Became unavailable during the evaluation process.

#### Evaluation Round 1

Table S2 in Multimedia Appendix 1 displays the score of each QUEST item in the first round of evaluations, categorized by reviewer, with apps ranked according to their mean total score. We explored potential correlations between the QUEST scores, Play Store ratings, and the scores of each certification body and found no significant correlations among any combination (data not shown). Agreement reached between reviewers was rather low, with most κ coefficient values below 0.6 and the intercorrelation coefficient between 0.50 and 0.67 (see Table S3 in Multimedia Appendix 1). The main reasons for discrepancy were attributed to the difficulty in finding the information needed to complete the QUEST scale.

#### Evaluation Round 2

Four additional experts were asked to update the app review on an Android device and were given a maximum of 45 minutes per app to find the information. At the time of this evaluation, 3 (15.8%) apps (*MyPlate*, *GetFit-Daily Meal Planner*, and *Uplyfe-Precision Nutrition*) were no longer available, and the remaining 16 (84.2%) apps were scored (see Table 3). One of the reviewers used an iOS device (Apple iPhone) due to unavailable Android devices. The 7 (43.8%) apps that received a score above 20 (out of a maximum of 28) either in the first evaluation or in the update were selected for further assessment.

Challenges were encountered assessing the items “authorship,” “attribution,” and “tone.*”* Some apps (particularly *LIFESUM*, *Freeletics Nutrition*, *Eat This Much-Meal Planner*, *LIFE Extend*, and *Second Nature*) lacked clear indications regarding authors and sources, necessitating external web searches to retrieve these data, and even then, this took considerable time. Additionally, reviewers noted that the “attribution” item could be easily altered to achieve higher scores; for example, referencing a source, even if it was a low-quality source or irrelevant to the app’s health information, could lead to a high rating, as long as it was a highly scored type of study. Subjectivity was also a significant factor in evaluating the “tone*”* item, leading to disparities in scores. Finally, some of the discrepancies were explained by the differences in app versions (eg, *Freeletics Nutrition, Fresh Well*, and *Second Nature* improved considerably between review rounds, coinciding with version changes, whereas the opposite was true for *HealthifyMe*). In the case of *HealthifyMe*, this (and the low subjective score) led to its exclusion from the third round, despite scoring above 20 points in the first round. Discussion about the subjective scores and the contribution by the reviewer scoring all the selected apps in this round led to a more structured scoring procedure in the third round.

#### Evaluation Round 3

All experts participating in the previous rounds of evaluation, except one who was no longer available for the task, took part in this round. At the time of this evaluation (early 2024), LIFE Extend was not available, so only 6 (31.6%) apps were included in the third round. Discrepancies in the scores were discussed among researchers.

Subjective scores were also given to the selected apps by all six reviewers (see Table S4 in Multimedia Appendix 1 and Table 3). The scores were discussed in a joint meeting, which led to the selection of the top 5 (8.3%) apps: *Second Nature, Freshwell, Yazio,* *LIFESUM*, and *MyNetDiary*. The scores given by each evaluator are included in Multimedia Appendix 1.

*Second Nature* was consistently the most highly scored app and is also endorsed by the National Health Service (NHS) of the United Kingdom. Its present version includes clear references to the evidence supporting its contents. Usability is good and improved by the inclusion in the update of interactive content, such as videos with advice and encouragement. There is a wide variety of dietary patterns to choose from, and recipes with visual backup are provided. Furthermore, most of the features are free to use. However, food registering is rather cumbersome, and the tone of the recommendations could be more cautious.

*Freshwell* was developed by two British general practitioners and is used by the NHS in the United Kingdom. Usability is good, with pictures, recipes, and explanations. Long-term goals are included, promoting behavior change. A classification of foods is included based on the type of dietary pattern that is promoted (low carb). Study references are provided to support this recommendation, and a disclaimer is provided stating that the app provides educational content and is not to be considered medical advice. People with chronic conditions are advised to ask their health care provider. Other health-promoting pieces of advice include reducing sugar intake, snacks, and alcohol. The subjective score penalized the low-carb type of dietary pattern on which the app is based, since reviewers considered that there is not sufficient evidence to promote it to the general public as the “default” dietary pattern [25].

*Yazio* has high usability due to likable aesthetics, ease of use, accessibility, and the challenges included in the app. The challenges, which promote behavior change, can be chosen by the user from a list, including quitting chocolate, sugar, sweets, fast food, etc. The app includes information about healthy eating, which is supported by scientific evidence, and the standard recommended diet is Mediterranean style. However, it also includes dietary patterns with less scientific evidence of benefit, such as intermittent fasting or the keto diet. In fact, intermittent fasting is a prominent feature of this app, including challenges on how long you can fast for. A disclaimer recommends not using this pattern in the long term and seeing a physician if that is the intention of the user.

*LIFESUM* has a visually pleasant interface and agile and intuitive navigation, both of which lead to good user experience. Different objectives can be set, and the app includes easy and advanced recipes, which help with organization and motivation for healthier eating, although all this is only available in the premium version. The app includes a score for easy comparison and progress tracking, but the criteria for the score are not transparent. Studies supporting the contents were not easily found and were not there for all the recommendations. Once again, the latter included intermittent fasting and keto diets. Minor bugs were identified in the form of nonfunctioning links.

*MyNetDiary* offers an attractive design, easy navigation, and a variety of resources to engage users. It provides information about different types of diets and highlights their key aspects. Examples of recipes for each type of diet are included with detailed descriptions. The content is supported by a trained specialist in the nutritional management of diabetes and other health problems. A diary function to track daily food intake is included, and data can be entered via text, audio, or barcode scanning. The premium version offers daily advice and feedback. Users can also access a nutrition blog written by the same specialist behind the app. Professionals can subscribe to connect with users interested in weight loss.

## Discussion

### Principal Findings

In this paper, we highlighted the need for a common evaluation framework to assess the quality of health apps and proposed a robust approach to this end. Our use case focused on apps promoting healthy eating. A total of 19 apps were included, which had already been positively assessed by dedicated certification bodies. The latter used a variety of evaluation criteria, and most did not include content quality among them. Following a rigorous, three-step evaluation process by seven experts, only 5 apps were recommended. The selection was based on a comprehensive assessment of the quality of their contents (using QUEST), as well as their usability and the use of behavior change techniques.

Health apps have emerged as promising tools to promote healthier lifestyles, due to their widespread accessibility, user friendliness, and cost-effectiveness [26-28]. However, the app market’s diversity and the lack of systematic evaluation processes leave consumers with limited tools to judge which apps are effective, safe, and suited to their needs.

To address this issue, several national and international actions have attempted to assess health app quality and safety [17,20-23]. Our investigation identified 28 initiatives dedicated to the evaluation of health apps, 5 including apps fulfilling our inclusion criteria. Although each of these initiatives uses distinct criteria for app evaluation, they all generally emphasize similar key factors, such as data security, privacy, ease of use, accessibility, usability, support for user-health care professional communication, personalization options, and capacity to induce behavior change. In a recent review, the importance of adherence to scientific evidence, as well as additional features (eg, gamification and cocreation of the app with health professionals and users), were supported [29]. Although a multitude of tools and scores have been proposed to evaluate different aspects of app quality, as of today, one single robust procedure or set of criteria does not exist [30-32], although some national and international evaluation efforts have been developed [33-35]. Additionally, all the certification bodies reviewed in this study have cautioned that they cannot guarantee the precision, quality, trustworthiness, and effectiveness of the health apps they assess, increasing uncertainty for the users.

Acknowledging these challenges, our study sought to evaluate nutrition-focused health apps using a tool aimed at the health information within the apps, assuming that health information based on evidence would support safety and effectiveness. We selected QUEST to assess the apps, since it has been specifically designed to evaluate health information and incorporates items such as attribution or study type to evaluate the quality of information sources. Additionally, it assesses items such as conflict of interest and tone to evaluate how information is presented to the user, where any sort of advertising is penalized. We found no significant correlations among the Play Store scores for the apps, the QUEST total mean score, or any of the certifier scores, probably because they assess different dimensions. QUEST is a robust tool for the assessment of content quality but does not address usability, engagement, or behavior change techniques, all factors known to influence the perceived quality of an app [11]. Therefore, the research team decided to complement this assessment with a subjective score, which also included these aspects. Such factors may influence the app store ratings, and many are directly evaluated by the certification bodies but are overlooked by QUEST. In contrast, Play Store scores are based on user opinions, which can be influenced not only by all these factors but also by popularity, endorsement by influencers, aesthetics, or alignment with their own nutritional preconceptions, which might not be backed by scientific evidence.

Furthermore, it is important to acknowledge that apps are dynamic and subject to constant updates. Although the latter can be beneficial if they enhance the quality of app contents, they also pose a challenge for app evaluation, as acknowledged by a recent consensus [36]. Thus, continuous reassessment would be necessary to account for the changes. Access to older app versions may not always be possible, and certain apps may become completely unavailable over time. Any app marketed as a health app should be subject to special scrutiny, particularly regarding collection of sensitive data and potential for harmful effects on users. Previously proposed improvements include establishing national lists of tested and trusted health apps, creating a catalog of health apps accessible to patients only if prescribed by professionals and guidelines for app developers toward evidence-based, unbiased, high-quality apps, with wider assessment requirements for higher-risk tools [14,33,36-39].

### Strengths and Limitations

Our work has several strengths that contribute to the field of health app evaluation. First, the adoption of QUEST to assess all the apps constitutes a rigorous approach for the evaluation of content quality. This systematic assessment of the content was complemented by a subjective evaluation by the panel of usability and behavior change techniques. Additionally, the study’s methodological approach, involving multiple, independent reviewers and the evaluation of a diverse range of apps, strengthens the validity and comprehensiveness of the results. Finally, the study’s alignment with the existing literature and national and international certification/assessment bodies highlights its relevance and potential impact on improving health app quality and user safety.

We acknowledge that the study also has some limitations. The small sample size of apps reviewed and their focus on nutrition-related interventions may limit the generalizability of our results, since they represent an incomplete picture of the overall quality and safety of available health apps. The decision to exclude apps that solely function as food diaries and calorie counters may be controversial. We chose apps offering recommendations, meal plans, and recipes, because we focused on the quality of the information provided. Nevertheless, we acknowledge that food diaries and calorie counting could also help the users become more aware of their eating habits and induce a change in behavior despite the lack of any other specific nutritional advice and therefore be considered a type of intervention. Another limitation is the subjective nature (eg, tone) or the scoring difficulty (eg, attribution) of some of the items in QUEST. Discrepancies in the first round of evaluations, reflected by rather low κ coefficient values, were mainly attributed to the fact that the information to score some items might be difficult to find in the app or might even be found behind a link and thus not be obvious. Hence, in the second round of evaluations using QUEST, we standardized the time spent on the assessment of each app. In addition, some aspects of app quality are overlooked by QUEST (eg, usability, engagement, and capacity to induce positive behavior changes). Thus, QUEST is not a comprehensive enough tool to judge app quality on its own, but it is complementary to other measures. Finally, to overcome these difficulties, several rounds of revision were made, which made the evaluation process longer than initially planned.

To summarize, during this review, we encountered several challenges. In the first place, numerous certification bodies were scanned for evaluated apps. Creating a common repository collecting their criteria and decisions would facilitate this task [40]. In addition, the rapid change in mHealth apps poses a challenge to the timeliness of evaluation. This might be mitigated by the development of living systematic reviews, which are continuously updated [39] or the use of rapid reviews [41] and evidence maps [42]. Lastly, there is currently no standardized procedure to evaluate app quality. To address this, we included what we considered a critical and often overlooked dimension in previous evaluation efforts: a thorough assessment of the quality of the health information provided by the apps using QUEST scoring.

### Conclusion

In conclusion, despite their previous evaluations by various certification bodies, only 5 of 19 apps promoting healthy eating met the quality standards set by our experts. Our study calls for enhanced scrutiny and regulatory measures to ensure that health apps meet rigorous standards of accuracy, reliability, and user safety. Guidelines for developers for the design of evidence-based, unbiased, high-quality health apps, as well as technological solutions for real-time monitoring of the apps, would address these challenges and improve reliability.

## Appendix

Multimedia Appendix 1 Initial search strategy, list of certifying bodies examined, scores given to apps by evaluators, interobserver concordance analysis, and subjective assessments by reviewers.

Multimedia Appendix 2 Preferred Reporting Items for Systematic Reviews and Meta-Analyses (PRISMA) checklist.

## Abbreviations

ABACUS: App Behavior Change Scale
GGD: *Gemeentelijke Gezondheidsdienst * (Municipal Health Service)
MARS: Mobile App Rating Scale
mHealth: mobile health
NHS: National Health Service
ORCHA: Organization for the Review of Care and Health Applications
PRISMA: Preferred Reporting Items for Systematic Reviews and Meta-Analyses
QUEST: Quality Evaluation Scoring Tool

## Data Availability

All relevant data are available for direct review within the paper.
