# Supplementary material for: The Landscape of Mobile Apps for Healthy Eating: Case Study for a Systematic Review and Quality Assessment
Source: JMIR Mhealth Uhealth. 2026 Jan 30;14:e68737. doi: 10.2196/68737 (PMC12905560; doi:10.2196/68737)
Supplement: Multimedia Appendix 1 [file mhealth_v14i1e68737_app1.docx]

**The landscape of mobile applications for healthy eating: a systematic review and quality assessment.**

Garlene Zamora Zamorano*, Alejandro Déniz García* et al. *Equivalent contribution

**Supplementary text describing search strategy**

**Initial Search Strategy**

The identification of applications was based on a structured search using two key documents that compile and analyze various initiatives related to health applications [references 11 and 13]. From these documents, a comprehensive list of the mentioned initiatives was created. Each initiative was then individually reviewed to examine its content and structure.

During this review, a notable heterogeneity among the initiatives was observed, allowing them to be classified into two main groups: (1) platforms offering pre-evaluated applications, used as databases for the search, and (2) those focused on normative aspects, standardization, and quality assessment.

The search for applications was conducted exclusively on platforms from the first group. Given the diversity in the approaches and structures of these platforms, the search used general terms related to the study’s focus—including *healthy eating*, *diet* and *healthy habits*—which were later adapted to the navigation logic, taxonomy, or filtering system of each initiative.

**Healthy Living Apps**

This initiative reported a collection of over 300 apps classified into five thematic categories: mental wellbeing, healthy eating, physical activity and sport, alcohol harm prevention, and tobacco prevention. It also offered filtering options by operating system (iOS or Android) and a keyword search engine.
For this review, we focused on the *healthy eating* category. A total of 22 apps were initially identified.

**MyHealthApps**

This platform allowed searches by both keyword and thematic categories. We used the terms such *nutrition*, *diet*, and *healthy eating* to identify relevant apps. Additionally, the 22 available categories were explored, with *Staying Healthy* proving to be the most relevant to the study’s objectives.
Within this category, apps were organized into subcategories such as *diet*, *lifestyle*, *preventive medicine*, and *weight/obesity*, among others. Each subcategory was systematically reviewed, resulting in the initial identification of 32 apps.

**ORCHA (Organisation for the Review of Care and Health Applications)**

This platform included a repository of over 3,500 health applications. The search was conducted using the following keywords, relevant to the study: *diet*, *healthy eating*, *nutrition*, *weight management* and *weight*. Each resulting app was reviewed individually.
Additionally, the thematic category *Healthy Living* was explored, which contained 312 apps organized into 16 subcategories, including *obesity*, *wellness*, *nutrition*, *weight loss*, *smoking cessation*, *sleep hygiene*, *relaxation techniques*, and *fluid intake*.
Priority was given to the *Nutrition* subcategory, where apps were further organized into functional categories such as *weight management*, *food diary*, *habit tracker*, *health diary*, *information*, *instant messaging*, and *home workout*. Each functional category was systematically reviewed, and 51 apps were initially identified.

**GGD AppStore**

This platform supported searches by thematic categories. Six main categories were identified: *Body Functions*, *Mental Well-being*, *Meaning*, *Quality of Life*, *Participation*, and *Daily Functioning*. For this review, the categories *Body Functions*, *Quality of Life*, and *Daily Functioning* were prioritized due to their relevance to the study’s objectives. Six applications were initially identified.

**Health Navigator**

This platform allowed app searches via an alphabetical index, through the *App Library Search* function, or by selecting representative images within the *App Library* section, which served as thematic categories. Fifty-one categories were identified, many of which focused on harmful habit prevention (e.g., *Alcohol Use*, *Quit Smoking*), treatment of specific health conditions (e.g., *Parkinson’s*, *Migraine and Headache*, *Multiple Sclerosis*, *Gout*, *Diabetes*), and general wellbeing.
For this review, the categories *Nutrition Apps*, *Fitness and Weight Loss*, and *Weight Control* were prioritized due to their relevance to the study’s objectives. Other complementary categories, such as *Healthy Ageing*, were also explored. Eight apps were identified.

It is important to note that in several of the reviewed initiatives, the same app could appear in multiple categories. In such cases, for the purposes of this study, each app was recorded only once. Additionally, the criteria were applied systematically across all platforms.

**Table S1.** Certifying bodies examined.

| **Organization/Initiative** | **Country** | **Link** |
| --- | --- | --- |
| 1. AppCheck | Germany | [ZTG AppCheck \| Die Informations- und Bewertungsplattform für Gesundheits-Apps](https://appcheck.de/) |
| 1. AppScript | United Kingdom | [AppScript - IQVIA](https://www.iqvia.com/locations/united-kingdom/solutions/nhs-solutions/appscript) |
| 1. Appsaludable | Spain | <http://www.calidadappsalud.com/distintivo/catalogo> |
| 1. AppSalut* | Spain | https://pasiona.com/proyectos-portfolio/appsalut/ |
| 1. Appteca | Spain | [APPs - Sociedad Española de Cardiología (secardiologia.es)](https://secardiologia.es/publicaciones/apps) |
| 1. BfArM DiGA* | Germany | https://www.bfarm.de/EN/Medical-devices/Tasks/Digital-Health-Applications/_node.html |
| 1. Dekra | France | https://www.dekra-certification.fr/certification-de-services/certification-applications-mobiles-et-sites-web-dekra-certification.html |
| 1. DiGA | Germany | [DiGA-Verzeichnis (bfarm.de)](https://diga.bfarm.de/de/verzeichnis) |
| 1. DIGI-HTA | Finland | https://www.oulu.fi/cht/digihealthhub/digi-hta |
| 1. ehealthsuisse | Switzerland | <https://www.e-health-suisse.ch/gemeinschaften-umsetzung/ehealth-aktivitaeten/mhealth.html> |
| 1. GGD AppStores | Netherlands | [GGD AppStore](https://www.ggdappstore.nl/Appstore/OverGGDappstore#:~:text=Het%20doel%20van%20de%20GGD%20AppStore%20is%20het,betrouwbare%20gezondheidsapps%20en%20websites%20%28zogenaamde%20E-Public%20Health%20toepassingen%29) |
| 1. Good practice guidelines on health apps and smart devices | France | [Haute Autorité de Santé - Good practice guidelines on health apps and smart devices (mobile health or mhealth) (has-sante.fr)](https://www.has-sante.fr/jcms/c_2681915/en/good-practice-guidelines-on-health-apps-and-smart-devices-mobile-health-or-mhealth) |
| 1. Health Navigator | New Zealand | <https://www.healthnavigator.org.nz/apps/a/app-library/> |
| 1. The Healthy Living Apps | Australia | <https://www.vichealth.vic.gov.au/media-and-resources/vichealth-apps> |
| 1. Helsenorge tools | Norway | [Verktøy - Helsenorge](https://tjenester.helsenorge.no/verktoy/09719787-02ea-445e-a166-e4c96e0b8533?verktoytype=1) |
| 1. HL7 International (Health Level Seven International) | United States | [HL7 Standards Product Brief - HL7 Consumer Mobile Health Application Functional Framework (cMHAFF), Release 1 \| HL7 International](https://www.hl7.org/implement/standards/product_brief.cfm?product_id=476) |
| 1. iPrescribeApps | United States | [iPrescribeApps - From clinical problem to mobile solution.](https://iprescribeapps.com/) |
| 1. iMedicalApps | United States | [iMedicalApps - Reviews of Medical apps & Healthcare Technology](https://www.imedicalapps.com/) |
| 1. iSYS Foundation | Spain | [Todas las APPS (fundacionisys.org)](https://www.fundacionisys.org/es/apps-de-salud/todas-las-apps) |
| 1. mHealthBelgium | Belgium | [All apps - mHealthBELGIUM](https://mhealthbelgium.be/apps) <https://mhealthbelgium.be/apps> |
| 1. MYSNS SPMS | Portugal | [MySNS Seleção – MySNS Comunidade (min-saude.pt)](https://mysns.min-saude.pt/mysns-selecao/) |
| 1. MyhealthApps* | United Kingdom | https://myhealthapps.net/ |
| 1. NEN | Netherlands | [Health and Welness apps (nen.nl)](https://www.nen.nl/en/health-and-welness-apps) |
| 1. NHS Health Apps Library* | Great Britain | <https://www.nhs.uk/apps-library/> |
| 1. One Mind PsyberGuide | United States | [Mental Health App Guide \| One Mind PsyberGuide](https://onemindpsyberguide.org/apps/) |
| 1. ORCHA (Organisation for the Review of Care and Health Apps) | United Kingdom | [ORCHA](https://appfinder.orcha.co.uk/) |
| 1. Ranked Curated Health Apps* | United States | <http://www.rankedhealth.com/> |
| 1. Southern Denmark MIndApps.dk | Denmark | [Appbibliotek - MindApps](https://mindapps.dk/appbibliotek/) |

*Not available in February 2024

**Table S2.** Scores given to each app by each evaluator for every element of the QUEST tool during the first evaluation round.

| **App** | **Auth.** | **Attr.** | **Study** | **Conf. int.** | **Curr.** | **Comp.** | **Tone** | **Total** | **Total Mean** |
| --- | --- | --- | --- | --- | --- | --- | --- | --- | --- |
| ***HealthifyMe*** | 2  1 | 9  9 | 2  2 | 3  3 | 2  2 | 1  1 | 3  3 | **22**  **21** | **21.5** |
| ***Freshwell*** | 0  2 | 6  9 | 2  2 | 6  6 | 2  2 | 0  0 | 3  3 | **19**  **24** | **21.5** |
| ***Yazio*** | 1  1 | 9  9 | 2  2 | 3  6 | 2  2 | 0  0 | 3  3 | **20**  **23** | **21.5** |
| ***Lifesum*** | 2  2 | 9  6 | 2  1 | 3  6 | 2  2 | 0  0 | 3  3 | **21**  **20** | **20.5** |
| ***Life Extend*** | 2  2 | 9  9 | 2  2 | 0  3 | 2  2 | 1  1 | 3  3 | **19**  **22** | **20.5** |
| ***MyNetDiary*** | 1  2 | 9  3 | 2  0 | 3  3 | 2  2 | 0  1 | 3  6 | **20**  **17** | **18.5** |
| ***Noom*** | 2  1 | 9  0 | 2  0 | 6  6 | 2  0 | 0  1 | 3  3 | **24**  **11** | **17.5** |
| ***GetFit*** | 0  0 | 0  9 | 0  2 | 6  6 | 0  1 | 0  0 | 3  6 | **9**  **24** | **16.5** |
| ***MyPlate*** | 2  2 | 3  9 | 0  1 | 3  3 | 2  0 | 0  0 | 0  3 | **10**  **18** | **14** |
| ***Fastic Fasting App*** | 2  2 | 3  3 | 1  0 | 6  6 | 2  0 | 0  0 | 3  0 | **17**  **11** | **14** |
| ***Uplyfe-Precision Nutrition*** | 0  0 | 3  9 | 0  2 | 3  3 | 2  2 | 0  0 | 0  3 | **8**  **19** | **13.5** |
| ***Second Nature*** | 1  1 | 6  3 | 1  0 | 3  3 | 2  0 | 1  0 | 3  0 | **17**  **7** | **12** |
| ***Fatsecret*** | 0  1 | 3  3 | 0  0 | 6  3 | 0  0 | 0  0 | 3  3 | **12**  **10** | **11** |
| ***Eat This Much*** | 1  0 | 6  0 | 0  0 | 3  3 | 2  0 | 0  1 | 3  3 | **15**  **7** | **11** |
| ***8fit Workout &MealPlanner*** | 1  1 | 6  3 | 2  0 | 0  0 | 1  0 | 0  0 | 3  3 | **13**  **7** | **10** |
| ***Dietbet*** | 1  1 | 3  3 | 0  0 | 3  3 | 0  0 | 0  0 | 3  0 | **10**  **7** | **8.5** |
| ***Freeletics Nut.*** | 1  1 | 0  0 | 0  0 | 0  3 | 0  0 | 0  1 | 6  3 | **7**  **8** | **7.5** |
| ***MyfitnessPal*** | 1  2 | 0  0 | 0  0 | 0  0 | 2  2 | 0  0 | 3  3 | **6**  **7** | **6.5** |
| ***Contador de calorías*** | 0  0 | 0  0 | 0  0 | 3  3 | 0  0 | 0  0 | 3  3 | **6**  **6** | **6** |

Abbreviations: Auth. Authorship, Attr. Attribution, Conf. Int. Conflict of interest, Curr. Currency, Comp. Complementarity.

**Table S3.** Inter-observer concordance analysis. Weighted kappa was used for the individual item scores and intra-class correlation coefficient for the total score.

| **QUEST EVALUATION** | **REVIEWERS 1 and 2**  **n = 8 apps** | **REVIEWERS 1 and 3**  **n = 7 apps** | **REVIEWER 2 and 3**  **n = 4 apps** |
| --- | --- | --- | --- |
| **Total Score**  (range 0 to 28) | 0.5 CI95% -0.29 – 0.88 | 0.67  CI95% 0.20 – 0.93 | 0.66  CI95% 0.11 – 0.97 |
| **Authorship**  (categories 0, 1 or 2) | 0.27 CI95% -0.27 – 0.82 | 0.46  CI95% 0.01-0.91 | 0.75  CI95% 0.36 – 1 |
| **Attribution**  (0, 3, 6 or 9) | 0.15  CI95% -0.34 – 0.65 | 0.27  CI95% -0.28 – 0.82 | 0.5  CI95% -0.21 – 1 |
| **Type of study**  (0, 1 or 2) | 0.25  CI95% -0.22 – 0.72 | 0.29  CI95% -0.37 – 0.94 | 0.33  CI95% -0.22 – 0.89 |
| **Conflict of interest**  (0, 3 or 6) | 0.52  CI95% 0.05 – 0.99 | 0.42  CI95% -0.13 – 0.97 | 1  CI95% 1-1 |
| **Currency**  (0, 1 or 2) | 0.57  CI95% 0.04 - 1 | Insufficient sample | 0.33  CI95% -0.22 – 0.89 |
| **Complementarity**  (0 or 1) | Insufficient sample | 0.09  CI95% -0.62 – 0.8 | 1  CI95% 1 – 1 |
| **Tone**  (0, 3 or 6) | -0.09  CI95% --0.27 – 0.09 | -0.11  CI95% -0.31 – 0.11 | 0  CI95% 0 – 0 |
| *ICC : intraclass correlation coefficient. **CI95% : 95% confidence interval. ***k: weighted kappa | | | |

**Table S4.** Subjective assessments by reviewers during the third round of evaluations

| **App** | **Reviewer** | **Quality of**  **content  (40%) 4 points** | **Easy of use/**  **user friendliness**  **(30%) 3 points** | **Promoting behaviour change**  **(30%) 3 points** | **TOTAL SCORE** |
| --- | --- | --- | --- | --- | --- |
| **Yazio** | 1 | 2 | 2.5 | 2.5 | 7 |
|  | 2 | 2 | 3 | 2.5 | 7.5 |
|  | 3 | 3 | 1 | 3 | 7 |
|  | 4 | 2.5 | 1.5 | 2.5 | 6.5 |
|  | 5 | 3 | 3 | 2.8 | 8.8 |
|  | 6 | 2.5 | 2.5 | 2.5 | 7.5 |
| **Averages** | | **2.5** | **2.3** | **2.6** | **7.4** |
| **MyNetDiary** | 1 | 3 | 2.5 | 2 | 7.5 |
|  | 2 | 1.9 | 2 | 2 | 5.9 |
|  | 3 | 3 | 1 | 2 | 6 |
|  | 4 | 2.5 | 1.5 | 1.5 | 5.5 |
|  | 5 | 3.8 | 3 | 3 | 9.8 |
|  | 6 | 2 | 2 | 2 | 6 |
| **Averages** | | **2.7** | **2.0** | **2.1** | **6.8** |
| **Lifesum** | 1 | 2 | 3 | 2.5 | 7.5 |
|  | 2 | 2 | 2 | 2.5 | 6.5 |
|  | 3 | 3 | 1 | 3 | 7 |
|  | 4 | 2.5 | 1 | 2 | 5.5 |
|  | 5 | 2.5 | 2.5 | 2.5 | 7.5 |
|  | 6 | 2 | 2.5 | 2 | 6.5 |
| **Averages** | | **2.3** | **2.0** | **2.4** | **6.8** |
| **Freeletics Nut** | 1 | 2 | 2.5 | 2 | 6.5 |
|  | 2 | 1.5 | 1 | 1 | 3.5 |
|  | 3 | 3 | 1 | 2.5 | 6.5 |
|  | 4 | 2 | 1 | 1.5 | 4.5 |
|  | 5 | 1.5 | 2 | 2 | 5.5 |
|  | 6 | 2.5 | 2.5 | 1.5 | 6.5 |
| **Averages** | | **2.1** | **1.7** | **1.8** | **5.5** |
| **Second Nature** | 1 | 3.5 | 2.5 | 2 | 8 |
|  | 2 | 3 | 2 | 2.5 | 7.5 |
|  | 3 | 3 | 3 | 3 | 9 |
|  | 4 | 3.5 | 2 | 2.5 | 8 |
|  | 5 | 3.9 | 3 | 3 | 9.9 |
|  | 6 | 3.5 | 2.5 | 2.5 | 8.5 |
| **Averages** | | **3.4** | **2.5** | **2.6** | **8.5** |
| **Freshwell** | 1 | 2 | 2.5 | 2 | 6.5 |
|  | 2 | 3 | 1.5 | 1.4 | 5.9 |
|  | 3 | 3 | 3 | 3 | 9 |
|  | 4 | 2 | 1.5 | 2 | 5.5 |
|  | 5 | 3.2 | 2.5 | 2.8 | 8.5 |
|  | 6 | 2 | 2 | 2 | 6 |
| **Averages** | | **2.5** | **2.2** | **2.2** | **6.9** |
